# Mobilization after thrombolysis (rtPA) within 24 hours of acute stroke: what factors influence inclusion of patients in A Very Early Rehabilitation Trial (AVERT)?

**DOI:** 10.1186/s12883-014-0163-6

**Published:** 2014-08-27

**Authors:** Linnéa Muhl, Jenny Kulin, Marie Dagonnier, Leonid Churilov, Helen Dewey, Thomas Lindén, Julie Bernhardt

**Affiliations:** 1 Linköpings University, Linköping, Sweden; 2 AVERT, Early Intervention Research Program, The Florey Institute of Neurosciences and Mental Health, Austin Campus, 245 Burgundy St, Heidelberg 3084, VIC, Australia; 3 The University of Melbourne, Melbourne, Australia; 4 Department of Neurology, Austin Health, Heidelberg, Australia; 5 The Centre of Brain Research and Rehabilitation, Gothenburg University, Gothenburg, Sweden

**Keywords:** Stroke, Very early mobilization, Post-stroke complications, Recombinant tissue plasminogen

## Abstract

**Background:**

A key treatment for acute ischaemic stroke is thrombolysis (rtPA). However, treatment is not devoid of side effects and patients are carefully selected. AVERT (A Very Early Rehabilitation Trial), a large, ongoing international phase III trial, tests whether starting out of bed activity within 24 hours of stroke onset improves outcome. Patients treated with rtPA can be recruited if the physician allows (447 included to date). This study aimed to identify factors that might influence the inclusion of rtPA treated patients in AVERT.

**Methods:**

Data from all patients thrombolysed at Austin Health, Australia, between September 2007 and December 2011 were retrospectively extracted from medical records. Factors of interest included: demographic and stroke characteristics, 24 hour clinical response to rtPA treatment, cerebral imaging and process factors (day and time of admission).

**Results:**

211 patients received rtPA at Austin Health and 50 (24%) were recruited to AVERT (AVERT). Of the 161 patients not recruited, 105 (65%) were eligible, and could potentially have been included (pot-AVERT). There were no significant differences in demographics, Oxfordshire classification or stroke severity (NIHSS) on admission between groups. Size and localization of stroke on imaging and symptomatic intracerebral heamorrhage rate did not differ. Patients included in AVERT showed less change in NIHSS 24 hours post rtPA (median change = 1, IQR (−1,4)) than those in the pot-AVERT group (median change = 3, IQR (0,6)) by the median difference of 2 points (95%CI:0.3; p = 0.03). A higher proportion of rtPA treated AVERT patients were admitted on weekdays (p = 0.04).

**Conclusion:**

Excluding a possible clinical instability, no significant clinical differences were identified between thrombolysed patients included in AVERT and those who were not. Over 500 AVERT patients will be treated with rtPA at trial end. These results suggest we may be able to generalize findings to other rtPA treated patients beyond the trial population.

## Background

Stroke is one of the leading causes of death in the developed world today and for those who survive the event, chronic disability is a frequent complication [1]. Best practice clinical guidelines recommend two key treatments for acute ischemic stroke: care in a specialized stroke unit and thrombolysis with recombinant-tissue plasminogen activator (rtPA) [2],[3]. Stroke unit care (SUC) improves outcomes such as physical independence and increases chances of survival after stroke [4]. The most specific and biologically powerful treatment for acute ischemic stroke is thrombolysis with rtPA given within the first 4.5 hours of ischemic stroke onset [5],[6]. However, thrombolysis is not devoid of side effects and patients are carefully selected [7],[8]. Prominent among concerns is the fear of secondary symptomatic intracranial hemorrhage (sICH), which is one major reason preventing an extension of rtPA treatment to a broader spectrum of patients [9],[10].

SUC is characterized by close monitoring of physiological and neurological parameters and possible complications, hydration and nutritional intake, and early rehabilitation [11],[12]. The initiation of rehabilitation has been suggested to be of importance both to prevent and treat complications [13] and to improve recovery of walking [14]. Although SUC improves outcomes after stroke [15], several components of SUC are ill-defined and early mobilization (out of bed activity training), often part of the early rehabilitation package, is one of these. Early mobilization is currently being tested as part of an international clinical trial, AVERT (A Very Early Rehabilitation Trial). AVERT is a large phase III randomized controlled trial testing whether very early mobilization (VEM) reduces death and disability at three months post stroke. VEM is defined as mobilization out of bed commenced within 24 hours of stroke onset and continued frequently thereafter. This intervention is performed by a physiotherapist or nurse. Participants randomized to the control arm undergo standard stroke unit care. As rtPA is part of standard care, patients treated with rtPA can be included in the trial if the physician allows [16].

Mobilization of thrombolysed patients concerns some clinicians, and rtPA protocols often include 24 hours of bed rest after treatment. In a previous case-crossover designed study with hypothetical case vignettes, we explored the factors likely to influence the decision to allow patients to mobilize early after treatment with rtPA. The study of fifty-four clinicians found that perceived risk of neurological decline, especially due to sICH; infection of unknown cause; severe chest infection; severe stroke (NIHSS >20); drowsiness and confusion were factors that significantly influenced the decision to mobilize [17]. In reality we know little about what influences these decisions in every day practice.

Twenty three percent of the 1898 patients recruited to AVERT to date have been treated with rtPA (n = 447). Over 500 patients treated with rtPA are expected to be recruited by trial end, approximately half of whom will be mobilized within 24 hours of stroke. These data may broaden our understanding of the benefits or harms of combining these two treatments. However, as inclusion in AVERT is dependent on physician approval, it is likely that included patients will be selected, thus limiting our ability to generalize findings to a broader stroke population. The aim of this study therefore, was to compare the characteristics of rtPA treated patients in the AVERT trial with those not included, and to explore the factors influencing inclusion in the trial.

We hypothesized that the use of rtPA would create a selection bias in the AVERT recruitment process and therefore that thrombolysed patients included in AVERT would be different from the ones not included in the trial.

## Methods

### Study design and subjects

A retrospective audit of patient records from our largest recruiting site, Austin Health, Melbourne, Australia, was conducted. Austin Health is a large metropolitan teaching hospital and the pioneer site for AVERT. Thrombolysed patients have been recruited on site since the beginning of AVERT. The audit was approved by the Austin Health Human Research Ethics Committee.

### Procedure

The first AVERT patient treated with rtPA was recruited at Austin Health in September 2007. All patients treated with rtPA at this site between September 2007 and December 2011 were identified from the rtPA register on the stroke unit and the Australian Stroke Clinical Registry (AuSCR) [18]. Patients recruited to AVERT from Austin Health were identified through the AVERT database; researchers were blinded to participants’ group allocation. Medical records were retrieved and data extracted by two assessors independently. Both assessors extracted data from the same 10 clinical cases to confirm reliability of the procedure. Brain images were reviewed electronically in consultation with a neurologist, to determine stroke size and localization and the presence of hemorrhagic transformation.

### Data of interest

We wanted to examine three core data themes that might influence inclusion in AVERT: 1) patient demographics and stroke characteristics; 2) rtPA administration information (stroke to needle time) and patient response to treatment at 24 hours post thrombolysis and 3) time and day of admission, which may influence trial recruitment due to the availability of recruiters. Demographics of interest included age, sex, marital status, living arrangements on admission and country of birth. Premorbid physical state was assessed by means of the modified Rankin Scale (mRS) [19], and past medical history was recorded. Time and date of stroke symptoms onset were recorded, and the clinical presentation of the stroke was described using the Oxfordshire Classification [20] and the National Institutes of Health Stroke Scale (NIHSS) [21] on admission. In cases of a missing NIHSS, the score was estimated by review of medical records by two NIHSS-certified assessors. Stroke size and localisation were recorded from CT/MRI scans and scored as large cortical, small cortical, hemispheric lacunar, brainstem, cerebellar, hemispheric large subcortical or other type of stroke. A large cortical infarction was defined as involving the cerebral cortex, deep white matter and/or basal ganglia while a small cortical infarction only involved the cerebral cortex. Time, date and type of all scans relevant to the admission were noted. The time and date of rtPA injection was recorded as well as blood pressure, blood glucose level, heart rate, respiratory rate, oxygen saturation and temperature on admission and 24 hours after thrombolysis. The NIHSS at 24 hours post rtPA was also recorded. Complications 24 hours after thrombolysis, such as hemorrhagic transformation, bleeding of other origin, infection or deep venous thrombosis (as noted in medical record) were recorded. Hemorrhagic transformation was defined as any blood seen in the brain on imaging, while sICH was defined as the presence of blood at any site in the brain on CT or MRI scan, in combination with clinical deterioration of 4 points or more in NIHSS score [22]. If performed, date and results of carotid Duplex ultrasound were recorded. Time and date of hospital admission were noted for all patients. In regard to AVERT inclusion criteria [16], weekend admission was defined as admission between Friday 4 pm and Sunday 12 noon.

### Data analysis

Following a heuristic approach, a sample size of 150 was estimated to be large enough to allow for the differentiation of the multiple independent variables in the planned regression analysis [23].

Patient demographics, past medical history and details about current stroke were summarised using descriptive statistics. We used the AVERT inclusion and exclusion criteria (Table 1) to divide the population into three groups: 1) patients included in AVERT after rtPA (AVERT), 2) potential AVERT participants (pot-AVERT), i.e., patients treated with rtPA who were eligible but not recruited into AVERT, and 3) patients who did not meet AVERT inclusion criteria.

**Table 1:** AVERT inclusion and exclusion criteria

**Inclusion criteria**	**Exclusion criteria**
• 18 years or older	• Premorbid modified Rankin Score of 3, 4 or 5
• Recruited within 24 hours of stroke onset	• Deterioration within first hour of admission, resulting in direct admission to ICU, a documented clinical decision for palliative treatment, or immediate surgery
• Admitted to stroke care unit	
• Conscious – reacts to verbal commands as a minimum	• Concurrent diagnosis of rapidly deteriorating disease (e.g. terminal cancer)
	• Unstable coronary or other medical condition judged by investigator to impose hazard to patient if involved in trial
	• Systolic blood pressure < 110 or > 220 mmHg
	• Oxygen saturation < 92% (±supplemental oxygen)
	• Resting heart rate < 40 or >110 bpm
	• Temperature > 38.5°C
	• Enrolled in another intervention trial

The differences between AVERT and pot-AVERT patients were examined either by Wilcoxon-Mann–Whitney Rank Sum test or Fisher exact test depending on the nature of underlying distributions. Corresponding effect sizes were estimated using Hodges-Lehmann nonparametric shift estimator and Odd Ratios respectively with corresponding 95% confidence interval. For all statistical testing, the threshold of p < 0.05 was used for determining statistical significance. No multiplicity correction was undertaken as we were prepared to tolerate higher values of Type I error in order to make sure no difference between the two groups were missed. We planned to conduct a multiple logistic regression, including those factors shown to be significantly different between groups in the invariable analysis. STATA ICv12 was used for all analyses of between group comparisons.

## Results and discussion

**Figure 1:**
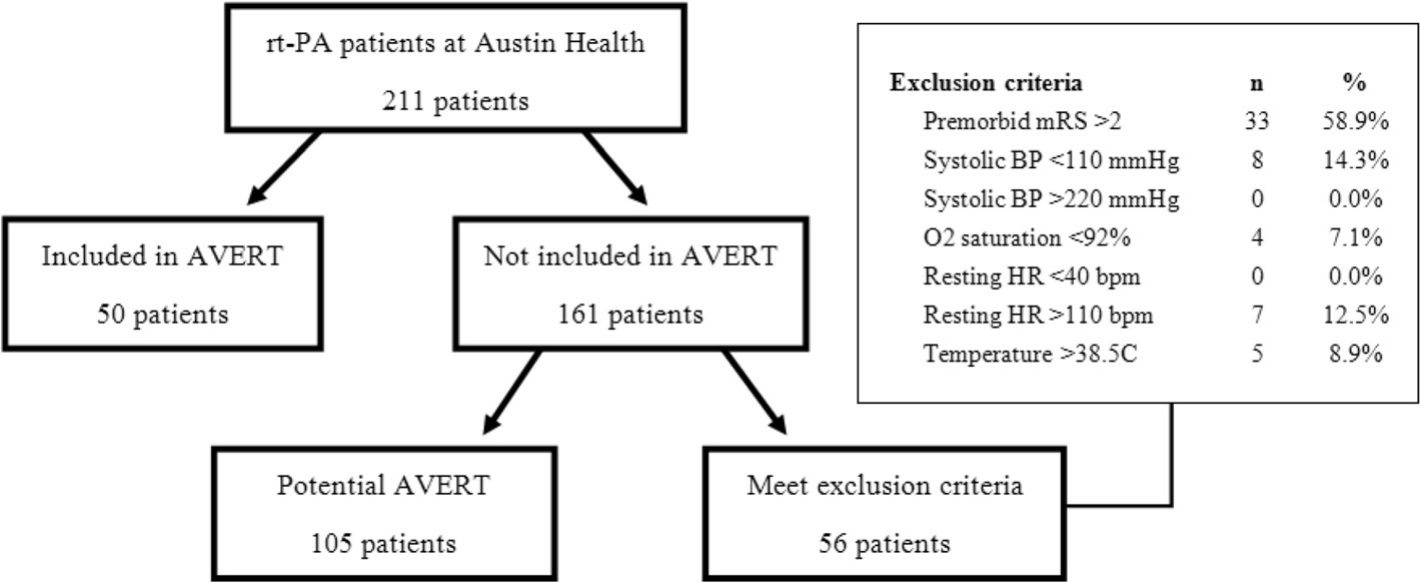
Patients selection: flow chart representing the patients review and classification regarding AVERT inclusion/exclusion criteria.

Between September 2007 and December 2011, 211 patients were treated with rtPA at Austin Health. Fifty (24%) were recruited to AVERT (here after known as “AVERT” group). Of the 161 patients not recruited, 105 (65%) were eligible according to the AVERT inclusion criteria and could potentially have been included in the trial (“pot-AVERT”) (Figure 1 and Table 2).

**Table 2:** Patients characteristics on admission

**Variable**	**potAVERT (n = 105)**		**AVERT (n = 50)**		Effect size* (95% CI)	**p**
Age (years)						
*Mean (±SD)*	73	(±13.6)	75	(±12.6)	2.25 (−1.18-5.60)	0.17
Sex						
*Female*	47	(45%)	23	(46%)	1.05 (0.54-2.06)	>0.99
Past medical history						
*Hypertension*	64	(61%)	38	(76%)	2.03 (0.96-4.28)	0.07
*Diabetes*	21	(20%)	16	(32%)	1.88 (0.89-4.01)	0.11
*High cholesterol*	47	(45%)	23	(46%)	1.05 (0.54-2.06)	>0.99
*Peripheral vascular disease*	4	(3.8%)	0	0	0 (0–2.00)	0.31
*Ischaemic heart disease*	22	(21%)	10	(20%)	.94 (0.42-2.15)	>0.99
*Angina*	6	(5.7%)	2	(4.0%)	0.69 (0–3.12)	>0.99
*Arthritis (lower limb)*	6	(5.7%)	4	(8.0%)	1.42 (0.41-4.94)	0.73
*Lower limb joint replacement*	4	(3.8%)	2	(4.0%)	1.05 (0–5.12)	>0.99
*Emphysema or COPD*	5	(4.8%)	1	(2.0%)	0.41 (0–2.74)	0.67
*Atrial fibrillation*	25	(24%)	14	(28%)	1.24 (0.59-2.65)	0.69
Premorbid mRS						
*Independent (mRS 0–2)*	105	(100%)	50	(100%)	N/A	>0.99
NIHSS On admission						
*Median (IQR)*	11	(6–17)	10.5	(6–18)	0 (−2-2)	0.91
Oxfordshire						
*TACI*	33	(31%)	24	(48%)		
*PACI*	48	(46%)	22	(44%)		0.09
*POCI*	7	(6.7%)	1	(2.0%)		
*LACI*	17	(16%)	3	(6.0%)		
Imaging						
*Large cortical*	56	(53%)	32	(64%)		
*Small cortical*	6	(5.7%)	1	(2.0%)		
*Hemispheric lacunar*	6	(5.7%)	3	(6.0%)		
*Brain stem*	6	(5.7%)	0	0		0.49
*Cerebellum*	1	(1.0%)	0	0		
*Hemispheric non cortical*	12	(11%)	4	(8.0%)		
*Other*	1	(1.0%)	0	0		

*Effect sizes expressed as H-L for continuous and OR for binary.

The overall rtPA treated population (44% women, 56% men) had a mean age of 74.3 years. A similar sex and age distribution were seen in both AVERT and pot-AVERT groups. Similarly, no significant difference was found between these two groups in terms of past medical history, NIHSS score on admission and Oxfordshire classification. All patients had a CT scan performed on admission and all patients, except one in pot-AVERT who died within 24 hours, had at least one follow up scan. No significant difference between the groups was found regarding infarction size or location. On admission the median NIHSS scores were similar for the AVERT (10.5, IQR 6–18) and pot-AVERT groups (11, IQR 6–17). A significant difference was identified in terms of NIHSS at 24 hours after rtPA between the AVERT and pot-AVERT groups, with median scores of 8.5 (IQR 5–14) and 5.5 (IQR 2–12) respectively, (p = 0.04; Table 3).

**Table 3:** Patients characteristics at time of thrombolysis and 24 hours after thrombolysis

**Variable**	**potAVERT (n = 105)**		**AVERT (n = 50)**		Effect size* (95%CI)	**p**
Stroke to needle (min)						
*Median (IQR)*	138	(108–156)	126	(108–156)	−1.2 (−15-13.2)	0.88
NIHSS						
At 24 hours						
*Median (IQR)*	5.5	(2–12)	8.5	(5–14)	2 (0–5)	0.04
Physiological data at thrombolysis						
*Hypertension*	31	(62%)	66	(63%)	0.96 (0.48-1.92)	>0.99
*Hypotension*	0	0	1	(2.0%)	N/A	0.32
*Hyperglycaemia*	3	(3.3%)	2	(4.6%)	1.41 (0–7.38)	0.66
*Hypoglycaemia*	0	0	2	(4.6%)	N/A	0.10
*Bradycardia*	13	(12%)	10	(20%)	1.77 (0.73-4.30)	0.23
*Tachycardia*	9	(8.6%)	4	(8.0%)	0.93 (0.29-3.01)	>0.99
*Arythmia*	24	(25%)	15	(33%)	1.48 (0.69-3.18)	0.32
*Rapid atrial fibrillation*	5	(4.9%)	3	(6.0%)	1.24 (0.31-4.92)	0.72
*Hypoxia*	1	(1.0%)	0	0	N/A	>0.99
Complications at 24 h post thrombolysis						
*Hemorrhagic transformation*	14	(13%)	10	(20%)	1.63 (0.68-3.91)	0.34
*sICH*	0	0	1	(2.0%)	N/A	0.32
*Bleeding other than cerebral*	12	(11%)	3	(6.0%)	0.49 (0.14-1.7)	0.39
*DVT*	1	(1.0%)	1	(2.0%)	2.12 (0)	0.54
*Infection*	9	(8.6%)	6	(12%)	1.45 (0.51-4.19)	0.56

*Effect sizes expressed as H-L for continuous and OR for binary.

Hypertension is defined as a blood pressure (BP) > 140/90 mmHg; hypotension: BP < 90/60 mmHg; hyperglycaemia; blood sugar level (BSL) > 13.5 mmol/L; hypoglycaemia: BSL < 4.0 mmol/L; bradycardia: heart rate < 60 beats per minute (bpm); tachycardia > 100 bpm; hypoxia: O2 saturation < 90%.

**Figure 2:**
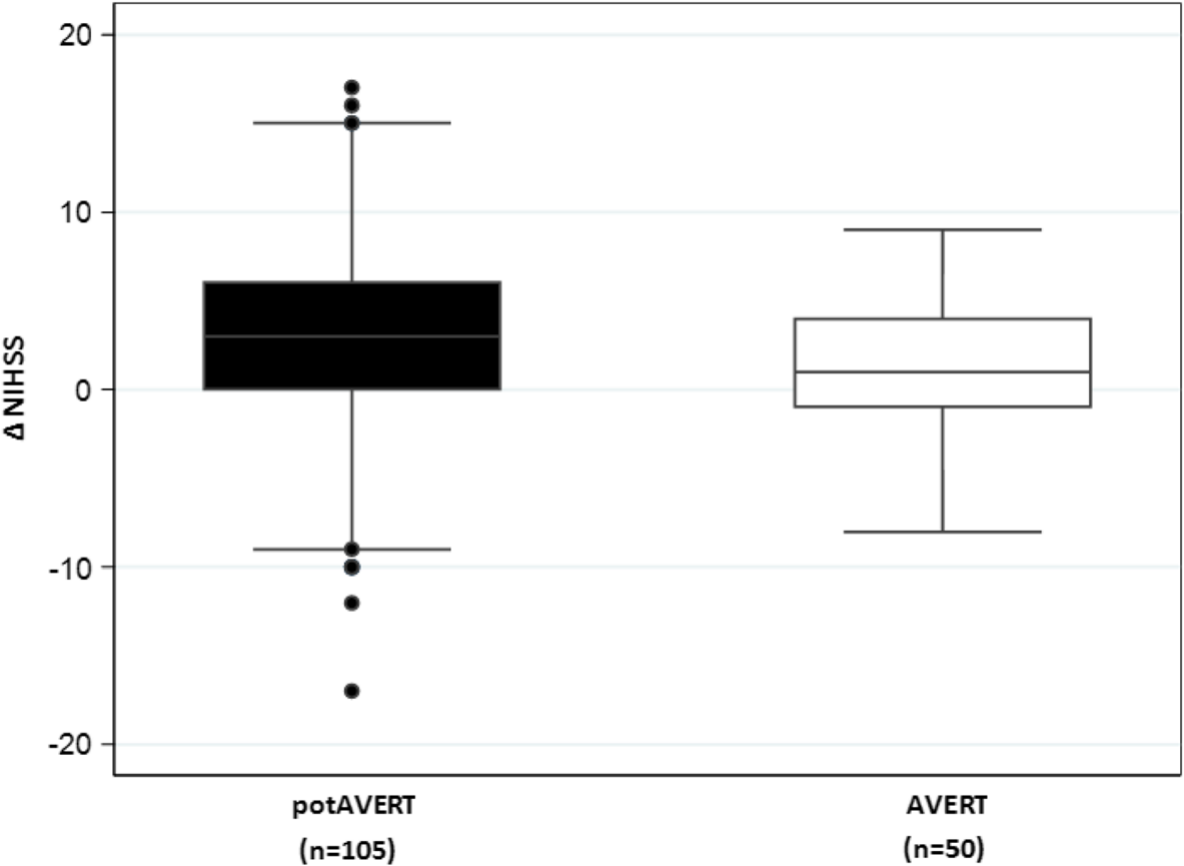
Change of NIHSS between groups: box plot comparing the distribution of the change in NIHSS from admission to 24 hours after thrombolysis (∆NIHSS) between the two subgroups.

Change in stroke severity, the difference between NIHSS on admission and NIHSS at 24 hours post rtPA (∆NIHSS), varied between groups (Figure 2). Patients included in AVERT showed less change in stroke severity; median change = 1, IQR (−1,4)) than those in the pot-AVERT group (median change = 3, IQR (0,6)) by the median difference of 2 points (95% CI:0–3; p = 0.03). In the pot-AVERT group, ∆NIHSS ranged from −17 (showing an improvement of 17 points) to +17. Fifty (48%) pot-AVERT patients improved by ≥4 points and 11 (10%) worsened by the same value. In contrast, in the AVERT group, ∆NIHSS varied from +9 to −8. Fourteen (28%) AVERT patients improved and 4 (8%) deteriorated by 4 points or more, the remainder of the patients did not change.

Physiological characteristics at rtPA administration and after 24 hours were not significantly different between the AVERT and pot-AVERT groups. Similar results were found for complications after thrombolysis. Neither hemorrhagic brain transformation, symptomatic intracranial haemorrhage, bleeding of other origin, deep venous thrombosis, nor infection varied between groups (Table 3). A regression analysis was not warranted.

Carotid Doppler ultrasound was performed in 84% of the AVERT patients and in 89% of the pot-AVERT patients. Thirty five percent of patients in each group had the examination performed within 24 hours. No significant differences were found in terms of presence of stenosis of any grade between the two groups. Of the patients who underwent Duplex ultrasound, 40% of AVERT and 32% of pot-AVERT had any degree of stenosis.

The rate of admission during the weekend was different between the groups (p = 0.04), with 12% of AVERT and 27% pot-AVERT patients admitted during that time.

In contrast to our original hypothesis, we found that thrombolysed patients recruited to the AVERT trial did not differ significantly at inclusion from those who were not included. Patients in both groups showed similar median NIHSS on admission. Instead, we identified a significant NIHSS change at 24 hours post thrombolysis, with AVERT patients on average changing less. The tighter distribution of ∆NIHSS score in the AVERT group could indicate that recruited patients were in a more stable state, without extremes in improvement or severe worsening within the first 24 hours post thrombolysis. This may have prompted physicians to agree to their recruitment in a clinical trial of mobilization. In contrast, 50% of the pot-AVERT patients improved or deteriorated by as much as 17 points on the NIHSS. Marked improvements in NIHSS might have prompted physicians to plan discharge early, while marked deterioration could have raised concerns regarding study inclusion. It is important to note that those recruited to AVERT (n = 50) were randomised to control or intervention, and that those randomised to the intervention (early mobilization) may have started within 24 hours of stroke onset. This may have contributed to the 24 hour NIHSS change. The majority of patients in AVERT are recruited to the trial at approximately 17 hours post stroke.

Based on the differences in NIHSS score before treatment and 24 hours after thrombolysis, patients may be divided into the “golden responders” with a great improvement in NIHSS score after thrombolysis, and the “non-responders” which include those with a severe stroke who keep deteriorating, or those with a severe stroke with little improvement after thrombolysis. Our results indicate that patients who show little improvement appear to be recruited in a higher proportion to AVERT. Such patients might be considered to have the possibility to regain more function in a rehabilitation trial, and are therefore recruited more frequently. The “golden responders” on the other hand, might be considered not to be in need of rehabilitation since they already do very well. Finally, patients with an extremely severe stroke and poor prognosis might be considered to be in a critical situation where rehabilitation will not have any benefit, or may even be harmful. These patients are generally not recruited to clinical trials.

In our previous study [17], we found that complications such as infection and sICH were factors that potentially influenced the decision to mobilize or prescribe bed rest after rtPA treatment. In this study, none of the explored complications were different between included patients and eligible, but not included, patients.

We included carotid Duplex timing and findings in this study because in some centres, vessel occlusion might be considered prior to allowing patients to get out of bed for the first time. In Australia, this is not the norm. As only 35% of the patients in both groups had the examination done within 24 hours, the carotid Duplex results were unlikely to impact the decision for early mobilization and therefore inclusion into the trial.

We elected to explore a range of factors that might influence inclusion in a clinical trial, based on previous research [17], clinical experience and our experience with recruitment challenges in this clinical trial. These may not be the only factors influencing recruitment to the trial. Physician opinion was not explored in this study. Physicians at the Austin do not follow a 24 hour rest in bed protocol and reported that they rarely, if ever, say no to eligible patients treated with rtPA participating in the trial. Nevertheless, unconscious bias may have been a factor. Participant refusal to participate in the trial and recruitment to another intervention trial might have contributed to the non enrolment of eligible thrombolysed patients into AVERT. AVERT screening logs show a refusal rate of less than 3% of approached patients. Therefore refusal to participate cannot be considered as the main reason for non recruitment of eligible thrombolysed patients.

A limitation of this study may be that in cases where the NIHSS was not recorded at the 24 hour post stroke time point, assessors were required to extract NIHSS from the clinical notes. However, it has been shown in previous studies that such a method has a high degree of validity and reliability [24],[25]. Since the same rating conditions were applied for all patients, regardless of their recruitment to AVERT, comparisons between the groups should not have been affected by this procedure.

Austin Health, the pioneer site for AVERT, is the highest recruiting centre for the trial. Even though our study focused on a single site, it provided a large sample of patients to allow exploration of selected factors. AVERT was running at 44 participating hospitals worldwide during the studied period, with rtPA treated patients recruited from 23 hospitals.

In terms of non patient related factors that could affect recruitment, we expected a difference between the groups regarding the time and day of admission. Indeed, thrombolysed patients admitted during the weekends were significantly less likely to be recruited to AVERT. The lower recruitment rate occurring during the weekends is explained by the unavailability of study personnel, who in this trial are staff physiotherapists and nurses.

## Conclusion

Excluding a neurological instability, no significant clinical differences were identified between thrombolysed patients included in AVERT and those who were not. These results suggest that efficacy results from rtPA treated patients in the completed trial (expected sample 500 patients) may be generalizable to the broader population of people treated with rtPA.

## Competing interest

All authors declare that they have no competing interest.

## Authors’ contribution

LM and JK have both contributed equally to this work. LM and JK extracted all the data, drafted the manuscript and participated in the statistical analysis. MD extracted part of the data, participated in the study design and helped to draft the manuscript. LC participated in the design of the study and performed the statistical analysis. JB and HD conceived of the study, and participated in its design and coordination and helped to draft the manuscript. TL supported LM and JK to participate in the research, and made critical comments during drafting of the manuscript. All authors read and approved the final manuscript.
